# Feasibility and safety of an exercise-based prehabilitation intervention before cancer surgery: a pilot randomised controlled trial

**DOI:** 10.1007/s00520-026-11356-9

**Published:** 2026-10-21

**Authors:** Declan T. Hennessy, Anna Wong Shee, Jonathan C. Rawstorn, Luke Evans, Stephen Brown, Matthew P. Wallen

**Affiliations:** 1 Institute of Health and Wellbeing, Federation University Australia, Victoria, Australia; 2 School of Exercise and Nutrition Sciences, Faculty of Health, Deakin University, Geelong, Australia; 3 Institute for Physical Activity and Nutrition, Deakin University, Geelong, Australia; 4 Grampians Health, Victoria, Australia; 5 Deakin Rural Health, Deakin University, Victoria, Australia; 6 Caring Futures Institute, College of Nursing and Health Sciences, Flinders University, Adelaide, South Australia Australia; 7 Faculty of Health Sciences and Medicine, Bond University, Robina, Queensland Australia

**Keywords:** Cancer, Exercise, Prehabilitation, Surgery, Feasibility

## Abstract

**Purpose:**

The study aimed to assess the feasibility and safety of an exercise-based prehabilitation intervention and explore preliminary clinical outcomes in breast, colorectal, and prostate cancer patients awaiting surgery in a large regional healthcare service.

**Methods:**

Patients with breast, prostate, or colorectal cancer scheduled for surgery were recruited at a single study centre and randomised (1:1) to prehabilitation or usual care. The intervention involved supervised aerobic exercise training 2–3 days per week before surgery. Usual care involved standard cancer treatment. Feasibility was assessed through recruitment, attendance, adherence, and attrition rates. Safety was evaluated via adverse event monitoring. Exploratory analyses evaluated group-by-time changes in cardiorespiratory fitness, health-related quality of life, and fatigue from baseline to the pre-surgery and post-surgery assessment timepoints, with 30-day post-operative outcomes (complications and length of stay) also collected.

**Results:**

Of 47 potentially eligible patients approached and screened, 23 were recruited and randomised, and completed baseline assessments. Twenty participants (prehabilitation *n* = 11, usual care *n* = 9) completed the pre- and T1 and T2 assessments. Participants attended an average of 5.25 ± 2.16 prehabilitation sessions. Attendance, adherence, completeness, and attrition rates were 91.3%, 82.6%, 85.7%, and 13%, respectively. Five adverse events were recorded in the prehabilitation group (fatigue, *n* = 3; muscular pain in the extremities, *n* = 2); all were grade 1. No significant group-by-time interactions were observed for cardiorespiratory fitness, health-related quality of life, or fatigue.

**Conclusions:**

Among participants who commenced prehabilitation, attendance, adherence, and completeness were 91.3%, 82.6%, and 85.7%, respectively. No serious adverse events were observed. Exploratory clinical outcome estimates were imprecise and did not support firm conclusions regarding intervention effectiveness. Recruitment limitations and the small heterogeneous sample also preclude broader conclusions regarding feasibility.

**Supplementary Information:**

The online version contains supplementary material available at https://doi.org/10.1007/s00520-026-11356-9.

## Introduction

Global estimates indicate that around 20 million new cancer diagnoses occur worldwide each year, with projections suggesting this burden could increase by approximately 77% by 2050 [1]. Breast, colorectal, and prostate cancers are among the most prevalent and rank among the four most commonly diagnosed cancers [1]. For many people diagnosed with these cancers, surgery remains the primary curative treatment [2–4]. However, many patients undergo surgery in a deconditioned state, often attributed to pre-existing comorbidities, prolonged inactivity, malnutrition, the systemic effects of cancer itself, or the iatrogenic effects of other cancer treatments [5, 6]. These factors contribute to reductions in key pre-operative physical qualities such as cardiorespiratory fitness, muscular strength, and functional exercise capacity, which are associated with poor peri- and post-operative outcomes including higher complications, delayed recovery, and prolonged hospital stay [7–9].

There is increasing focus on pre-operative strategies aimed at optimising physical qualities, psychological health, and quality of life ahead of cancer surgery [10]. Prehabilitation leverages the time from diagnosis to surgery to implement one or a combination of exercise, nutrition and psychology and behaviour change interventions in preparation for surgery, which have shown to enhance physiological reserve and resilience prior to known surgical stress [8, 10]. Exercise-based interventions delivered in the pre- and peri-operative periods have shown potential to improve cardiorespiratory fitness and functional exercise capacity, with evidence suggesting possible benefits for patient-reported fatigue [10–12]. However, these findings have not been consistent across trials, with some studies reporting no improvements in cardiorespiratory fitness or self-reported physical recovery [13, 14]. Reported effects on health-related quality of life, post-operative complications, and length of hospital stay remain variable across studies [10–12]. Recent systematic reviews indicate that prehabilitation may improve selected short-term post-operative and patient-reported outcomes. However, the magnitude and consistency of these effects vary according to intervention composition and surgical population, with evidence for unimodal exercise prehabilitation remaining uncertain across several cancer types [15–17].

Most existing prehabilitation trials have been conducted in metropolitan tertiary hospital centres and within relatively homogeneous cancer cohorts. This potentially limits the understanding of its effects in rural and regional settings where clinical services manage cohorts that are typically heterogeneous with differing disease trajectories and treatment pathways. Patients may also differ in baseline clinical characteristics, comorbidity burden, and disease presentation compared with metropolitan counterparts [18]. However, more recently, remote and hybrid exercise delivery may offer alternative approaches to reducing travel burden and improving access to prehabilitation for patients living in regional areas [19]. Within this trial, breast, colorectal, and prostate cancers were included to reflect the range of common surgical cancer populations managed within the regional health service and to evaluate whether a single prehabilitation model could be delivered across multiple cancer streams.

The aims of the present report of the PReOperaTive Exercise Therapy for patients with cancer (PROTECT) study were to (1) investigate the feasibility and safety of a structured exercise-based prehabilitation intervention in people diagnosed with breast, prostate, or colorectal cancer ahead of surgery in a regional healthcare service in Australia; and (2) explore the preliminary effects of the intervention on cardiorespiratory fitness, health-related quality of life, fatigue, and post-operative outcomes.

## Methods

### Trial design

This was a prospectively registered pilot randomised controlled trial (14/02/2019—ACTRN12619000214134) reported in accordance with Consensus on Exercise Reporting Template (CERT) and Consolidated Standards of Reporting Trials (CONSORT) guidelines [20, 21]. A pilot design was selected to evaluate uncertainties regarding recruitment, intervention delivery, attendance, adherence, and safety across three cancer streams within the short and variable pre-operative period in a regional healthcare service [22–24]. The trial used a two-arm parallel design, with participants randomised in a 1:1 ratio to the prehabilitation or usual care group. Grampians Health and St John of God Human Research Ethics Committee (46280) and the Federation University Human Research Ethics Committee (E19-001) prospectively approved the trial protocol. Written informed consent was obtained prior to any study-related activity. The study was conducted in accordance with the Declaration of Helsinki applicable at the time of study conduct [25].

Several differences between the trial registration and the study as conducted and reported should be noted. The registered trial included an effectiveness objective supported by a ventilatory threshold-based non-inferiority sample-size calculation; however, because the planned recruitment target was not achieved, the present report prioritises feasibility and safety, with clinical outcomes interpreted as exploratory. This revised reporting focus followed the recruitment shortfall and was not based on the direction or magnitude of the observed clinical outcomes. The comparator condition, intervention delivery format, and blinding differed from aspects of the registered description; the procedures delivered are described below. Intervention sessions were delivered individually, initially because of low concurrent enrolment and subsequently because of COVID-19 restrictions. Accelerometry and blood samples were collected as planned; only baseline accelerometry data are reported in the present manuscript, while biomarker analyses are not included.

### Participants

Patients were eligible to participate if they were (1) aged 18 years and older; (2) diagnosed with breast, prostate, or colorectal cancer and scheduled for surgery ≥ 2 weeks from enrolment; and (3) medically approved to undertake supervised exercise. Exclusion criteria included (1) any absolute contraindications to perform exercise testing per the American College of Sports Medicine guidelines [26]; (2) any adverse event during the initial cardiopulmonary exercise test (CPET) that indicated it would be unsafe to participate in the intervention; and (3) a diagnosis of metastatic disease.

All participants were recruited, enrolled, and assessed at the study centre. Participants were identified through weekly multidisciplinary meetings and outpatient clinics. Eligible patients were approached and invited to participate in the clinics, then screened, consented, and enrolled by an onsite medical oncologist on a separate day.

### Study procedures

Study assessments were conducted at three time points: baseline (T1), pre-surgery (T2), and 30 days post-surgery (T3). Prior to assessments, participants were required to continue all regular medications and refrain from any vigorous physical activity, caffeine, and alcohol for at least 36 h. Participants attended all assessments following an overnight fast (> 8 h), during which outcomes including cardiorespiratory fitness, health-related quality of life, and fatigue were assessed.

Blood samples were collected from the antecubital vein to assess immune and inflammatory biomarkers, and participants were also provided with an accelerometer (ActiGraph GT9X) to monitor and measure activity behaviours. These were collected as planned; only baseline physical activity data are reported in the present manuscript, while biomarker analyses are not included.

### Usual care

The usual care group continued to receive standard medical and allied health care at the study centre. During the trial, Grampians Health (previously Ballarat Health Services) did not have a standardised prehabilitation pathway within cancer care, and medical oncologists made referrals based on clinical need. Referred patients could receive an initial assessment with an Accredited Exercise Physiologist (AEP) or physiotherapist. The type (e.g. 6MWT) and number of outcome measures assessed were at the discretion of the treating clinician. Patients could then attend supervised group-based exercise sessions which may have included a home exercise program. Where supervised sessions were not practical, patients may have been provided with a home exercise program to complete independently. However, during this trial, there were no known referrals within the prehabilitation timeframe in the usual care group.

### Prehabilitation intervention

In addition to usual care, participants randomised to the prehabilitation group received an individualised aerobic exercise training programme. This involved one supervised exercise training session performed every 2 to 3 days until 48–72 h before their scheduled surgery. All exercise sessions were delivered one-on-one and on-site by an AEP. Individual delivery initially reflected the small number of participants enrolled concurrently and was subsequently maintained because of COVID-19 restrictions within the hospital.

Each session lasted 45–60 min, including a 5-min warm-up and a 5–10-min cool-down, and consisted of at least 30 min of continuous aerobic exercise performed on a cycle ergometer. All participants commenced training at 40% of the heart rate reserve (HRR) based on the resting and maximum heart rate achieved during the baseline CPET. As participants became accustomed to the exercise prescription and it was identified that they no longer reached the lower target intensity anchors confirmed by heart rate and self-reported exertion, the prescribed intensity was increased by 10% HRR every three sessions. Intensity was titrated within the 40–70% HRR range, corresponding to a rating of perceived exertion (RPE) of 11–16 (‘fairly light’ to ‘hard’) [27].

### Outcomes

#### Primary outcomes

##### Feasibility

Feasibility was assessed through recruitment (% of eligible participants enrolled), attendance (% of prescribed sessions attended), adherence (% of exercise sessions completed in full), completeness (% of sessions in which participants achieved at least 90% of prescribed HRR target zones), and attrition (% of participants lost to follow-up). Formal feasibility thresholds and progression criteria were not specified a priori. Accordingly, feasibility outcomes were reported descriptively and were not used to classify the intervention or trial procedures as meeting or failing predefined success criteria. Supervised exercise training sessions were evaluated for exercise adherence. During these sessions, attendance was defined as presence at scheduled exercise training sessions, while adherence was defined as completion of the prescribed exercise training protocol, including documentation of early session termination and reasons for non-compliance. Furthermore, attrition was reported at T2 as the proportion of randomised participants who did not complete the relevant follow-up assessment.

##### Safety

An adverse event was defined as any untoward or unfavourable medical occurrence in a participant, which may or may not be related to the intervention. Serious adverse events were defined as an adverse event that results in either death, a life-threatening situation, hospitalisation or prolongation of existing hospitalisation, persistent or significant disability/incapacity, or other important medical events (based on assessment by the study medical doctors). All adverse events and serious adverse events were graded using the Common Terminology Criteria for Adverse Events (CTCAE) Version 5.0 [28]. Adverse events and serious adverse events were gathered during the intervention period and confirmed by the study medical doctor. The severity of adverse events included mild (grade 1), moderate (grade 2), severe (grade 3), and life-threatening (grade 4), and death (grade 5). The adverse events were reported as a rate relative to exercise exposure, calculated as the number of adverse events per total hours of supervised exercise completed during the intervention period.

##### Exploratory outcomes

Cardiorespiratory fitness, health-related quality of life, fatigue, and post-operative outcomes were collected as exploratory measures to assess the completeness and practicality of outcome collection and to provide preliminary estimates of direction, magnitude, and variability to inform the design of future adequately powered trials.

##### Cardiorespiratory fitness

Before the CPET commenced, a resting ECG (Mortara ELI 230, Welch Allyn, USA), body mass (kg), and resting blood pressure (SphygmoCor XCEL, AtCor Medical Pty. Ltd., Australia) were obtained to ensure the participant was safe to exercise. Once cleared for exercise by the study oncologist and deemed eligible, a supervised, symptom-limited CPET was conducted on an electronically braked cycle ergometer (E100 ergometer, COSMED, Italy), following international guidelines [29, 30]. Gas analyses were calibrated prior to each test in accordance with manufacturer recommendations (two-point calibration against room air and a known gas mixture containing 5% carbon dioxide, 15% oxygen, and balance nitrogen).

Following 3 min of seated rest and 3 min of unloaded cycling, participants commenced an individualised incremental ramp test calculated using the Wasserman equations [29] until either (1) volitional fatigue, (2) a cadence drop below 50 RPM, or (3) accepted medical indications for test termination [26]. Breath-by-breath ventilatory gas exchange was measured (Metalyzer 3B, Cortex, Germany), including oxygen uptake (V̇O_2_), carbon dioxide production (V̇CO_2_), and minute ventilation (V̇E).

The ventilatory threshold (VT) was derived using the modified V-slope method and confirmed via the ventilatory equivalents method [31]. The average of the highest consecutive 20-s V̇O_2_ values was determined as peak V̇O_2_ [32]. The VT and peak V̇O_2_ were expressed in relative (mL kg^−1^ min^−1^) and absolute (L min^−1^) terms, and the VT was also reported as a percentage of peak V̇O_2_. The work rates (W) corresponding to the VT and peak V̇O_2_ were also recorded, along with the time to peak V̇O_2_, and the respiratory exchange ratio and rate pressure product obtained at peak exercise. The VE/V̇CO_2_ slope was calculated using the formula: V̇E (L min^−1^) = *m* (V̇CO_2_, L min^−1^) + *b*, where *m* = V̇E/V̇CO_2_ slope and *b* = constant.

##### Health-related quality of life

The Functional Assessment of Cancer Therapy – General (FACT-G) questionnaire was used to assess self-reported health-related quality of life over a 7-day period, which included domains related to physical, social/family, emotional, and functional well-being [33]. Scores from all domains were summed to provide a total score, with higher scores indicating better perceived well-being.

##### Fatigue

Fatigue was quantified via the Functional Assessment of Chronic Illness Therapy–Fatigue (FACIT-F) questionnaire [34], which consists of 13 items assessing fatigue severity and its impact on daily activities and functioning over the previous week. Higher scores on the FACIT-F represent lower levels of fatigue and better functioning.

##### Post-operative outcomes

All post-operative complications occurring within 30 days of surgery were recorded at the individual-event level, with each complication graded using the Clavien–Dindo classification system [35]. Complications were categorised by the severity of required treatment, ranging from grade 1 (no intervention) to grade 5 (death). Length of hospital stay was defined as the total elapsed time in days from formal inpatient admission to discharge or death. The study was not statistically powered to formally assess differences in post-operative outcomes; therefore, results are presented descriptively.

##### Sample size

The prespecified sample-size calculation reflected the original effectiveness objective and was based on VT derived from CPET. We selected the VT derived from CPET because higher pre-operative values have been associated with a lower risk of post-operative morbidity following colorectal and rectal cancer surgery [36, 37]. The prespecified calculation used a non-inferiority margin of 2.0 mL·kg⁻^1^·min⁻^1^, informed by previous supervised exercise research [38], and an assumed standard deviation of 1.8 mL·kg⁻^1^·min⁻^1^. With 90% power and a one-sided significance level of 2.5%, the estimated minimum sample was 38 participants. The recruitment target was increased to 50 participants to allow for attrition. As the required sample size was not achieved, no non-inferiority conclusion was drawn.

##### Randomisation

Following completion of the baseline assessments, participants were stratified by primary cancer diagnosis and randomly allocated in a 1:1 ratio to the prehabilitation or usual care group. The allocation sequence was computer-generated using variable block sizes of two, four, or six by an individual external to the investigation. Group allocation was revealed after completion of the baseline assessments. Because of the nature of the intervention, participants and intervention staff were not blinded to group allocation. Outcome assessors, including those determining cardiopulmonary exercise test outcomes, and the data analysts were not blinded.

##### Statistical methods

Statistical analyses were conducted using STATA/SE 15.1 [39]. Analyses of the clinical outcomes were exploratory and were not intended to provide definitive tests of intervention effectiveness. Normality and data distribution were inspected visually using histograms and scatterplots. Baseline characteristics are reported descriptively as group means ± standard deviations (SDs) as appropriate [40]. Continuous outcomes of both groups were compared using random effects mixed modelling with Tukey post hoc analyses, with time (T1, T2, and T3) and group allocation (intervention or usual care) as fixed factors. Sample sizes varied across assessment timepoints according to outcome availability. Standardised effect sizes were calculated using Hedges’ *g* for the CPET-derived outcomes, with corresponding 95% confidence intervals, and are presented in Supplementary Material 1. Given the small sample size that was achieved, effect sizes were not categorised using conventional magnitude thresholds and were interpreted cautiously.

## Results

**Fig. 1:**
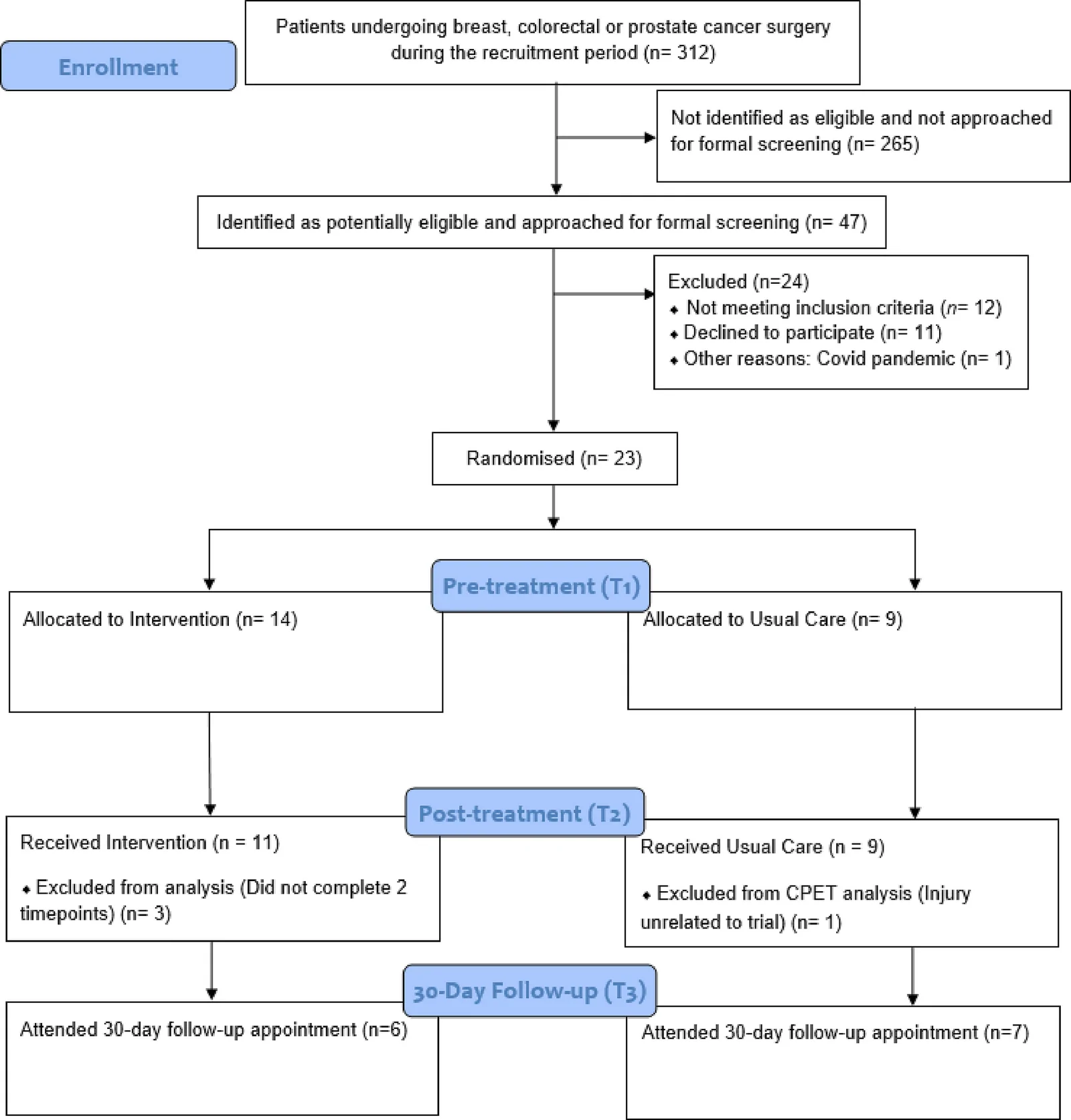
CONSORT flow diagram of participant recruitment and retention including reasons for dropouts

During the recruitment period, 312 patients with breast, colorectal, or prostate cancer underwent surgery with curative intent at the study centre. Forty-seven patients were identified as potentially eligible and approached for formal screening. Of these, 23 participants were enrolled and randomly assigned to either the intervention group (*n* = 14) or the usual care group (*n *= 9; Fig. 1), corresponding to 48.9% of approached patients and a recruitment rate of 1.92 participants per month. Reasons for non-enrolment included not meeting the study inclusion criteria (*n* = 12), declining participation (*n *= 11), or COVID-19-related issues (*n* = 1). Between T1 and T2, three participants were lost to follow-up due to COVID-19 public health restrictions. Consequently, 11 participants in the intervention group and nine in the usual care group completed the first two assessments (T1 and T2).

### Recruitment

Recruitment commenced in October 2019, paused in March 2020 due to the COVID-19 pandemic, resumed in March 2021, and concluded in September 2021 (Table 1).

**Table 1:** Baseline characteristics of the intervention and usual care groups

Participants’ characteristic	Usual care (*n* = 9)	Intervention (*n* = 11)
Age (years)	55.8 ± 12.5	59.3 ± 8.5
Females, *n*	5	5
Height (cm)	170.1 ± 11.2	172.0 ± 8.3
Body mass (kg)	95.7 ± 17.6	86.6 ± 14.3
BMI (kg m^−2^)	33.1 ± 5.3	29.3 ± 4.4
Resting RPP (mmHg bpm^−1^)	10,111 ± 1427	9119 ± 1337
Cancer type		
Breast, *n*	5	3
Colorectal,* n*	2	3
Prostate, *n*	2	5
Neoadjuvant chemotherapy, *n*	4	2
Moderate to vigorous physical activity (min week^−1^)*	141 ± 55.6	94 ± 42.2

*BMI* body mass index, *RPP* rate pressure product

*Data available for usual care, *n* = 7; and intervention, *n* = 11

### Baseline data

#### Feasibility

For the intervention group, the median number of exercise sessions was 5 (range 3–11). Exercise training attendance and adherence were 91.3% and 82.6% among the 11 participants who completed training from T1 to T2. The median time from study enrolment to surgery was 26.5 days (IQR 17.5–55.5). The median time from T1 to surgery was 18.5 days (IQR 14.5–37). In addition, the exercise intervention completeness was 85.71%, and study attrition was 13.0% at T2.

#### Safety

There were five adverse events recorded among four intervention participants (36%) during 63 total hours of exercise prehabilitation. This equated to one adverse event every 12.6 h of exercise. All events were classified as grade 1 and included fatigue (*n* = 3) and muscular pain in the extremities (*n* = 2). No adverse events precluded continued participation. There were no serious adverse events.

### Exploratory outcomes

Table 2 presents the exploratory cardiorespiratory fitness, health-related quality of life, and fatigue outcomes across the study period. Given the small achieved sample, these estimates were imprecise and were not interpreted as definitive evidence of intervention effectiveness.

**Table 2:** Cardiorespiratory fitness, health-related quality of life, and fatigue outcomes across multiple timepoints with groups and time interactions

Variable	Usual care *(mean* ± *SD)*			Intervention* (mean* ± *SD)*			Overall model *P* values		
	T1 (*n* = 8)	T2 (*n* = 8)	T3 (*n* = 7)	T1 (*n* = 11)	T2 (*n* = 11)	T3 (*n* = 6)	Group	Time	Group × time
Time to VT (s)	773 ± 215	833 ± 250	697 ± 114	741 ± 201	759 ± 115	593 ± 81	0.18	0.07	0.85
Absolute VT (L min^−1^)	1.2 ± 0.5	1.2 ± 0.5	1.1 ± 0.3	1.1 ± 0.4	1.1 ± 0.3	0.8 ± 0.2	0.16	0.22	0.90
Relative VT (mL kg^−1^ min^−1^)	12.8 ± 4.5	12.7 ± 4.0	11.4 ± 2.6	12.8 ± 4.2	13.4 ± 4.0	10.4 ± 3.3	0.94	0.29	0.85
Time to peak (s)	996 ± 181	1075 ± 249	984 ± 143	965 ± 149	1038 ± 98	792 ± 163	0.07	0.03	0.34
Absolute peak V̇O_2_ (L min^−1^)	1.7 ± 0.5	1.7 ± 0.5	1.5 ± 0.5	1.6 ± 0.4	1.6 ± 0.3	1.1 ± 0.4	0.15	0.04	0.58
Relative peak V̇O_2_ (mL kg^−1^ min^−1^)	18.0 ± 5.1	17.6 ± 4.2	15.6 ± 2.6	19.1 ± 5.0	19.3 ± 5.0	14.0 ± 5.2	0.76	0.06	0.59
Peak RER	1.09 ± 0.13	1.13 ± 0.12	1.17 ± 0.06	1.13 ± 0.08	1.16 ± 0.12	1.11 ± 0.11	0.87	0.50	0.44
V̇E/V̇CO_2_ slope	29.2 ± 3.7	28.6 ± 3.4	29.9 ± 2.8	32.3 ± 3.6	32.2 ± 4.8	33.6 ± 3.5	0.01	0.61	0.96
Peak workload (W)	113.8 ± 31.1	116.3 ± 30.7	100 ± 23.1	104.5 ± 22.5	117.3 ± 22.4	75 ± 24.3	0.14	0.01	0.38
	T1 (*n* = 9)	T2 (*n* = 9)	T3 (*n* = 7)	T1 (*n* = 11)	T2 (*n* = 11)	T3 (*n* = 6)			
FACT-G	78.7 ± 9.7	77.3 ± 12.2	73.6 ± 13.9	78.6 ± 9.6	78.2 ± 10.3	69.4 ± 17.1	0.75	0.19	0.82
Physical well-being	86.3 ± 9.0	85.7 ± 9.5	78.4 ± 13.8	87.3 ± 11.0	88.8 ± 8.2	75.8 ± 10.6	0.86	0.01	0.73
Social/family well-being	81.2 ± 13.0	78.1 ± 15.2	73.9 ± 13.1	80.3 ± 21.7	77.0 ± 20.6	68.9 ± 29.2	0.66	0.38	0.94
Emotional well-being	76.9 ± 13.4	75.5 ± 18.2	79.2 ± 17.4	75.2 ± 21.6	78.6 ± 16.4	74.7 ± 21.6	0.84	0.98	0.82
Functional well-being	70.0 ± 15.7	69.4 ± 16.8	63.4 ± 17.9	71.1 ± 10.7	68.5 ± 16.1	59.2 ± 29.6	0.78	0.29	0.91
FACIT-F	67.8 ± 22.8	71.4 ± 21.3	71.4 ± 18.4	80.7 ± 12.8	83.2 ± 9.3	63.7 ± 22.3	0.25	0.29	0.19

*VT* ventilatory threshold, *V̇O*_*2*_ oxygen uptake, *V̇CO*_*2*_ carbon dioxide production, *W* watts, *RER* respiratory exchange ratio, *FACT-G* Functional Assessment of Cancer Therapy-General, *FACIT-F* Functional Assessment of Chronic Illness Therapy-Fatigue

Within 30 days of surgery, six intervention participants and four usual care participants experienced at least one post-operative complication. Most recorded complications were Clavien–Dindo grade I, and one grade III complication occurred. The median length of hospital stay was 4 days in both groups, and one 30-day readmission was recorded. Given the small and heterogeneous cohort, these outcomes are presented descriptively (Table 3).

**Table 3:** Descriptive 30-day post-operative outcomes by study group

	Intervention (*n* = 11)	Usual care (*n* = 9)	Total (*n* = 20)
Patients with at least one post-operative complication, n	6	4	10
Recorded post-operative complication events by Clavien–Dindo grade,* n*			
Grade I	8	3	11
Grade II	2	2	4
Grade III	1	0	1
Grade IV	0	0	0
Median length of hospital stay (range)	4 (1–12)	4 (2–9)	4 (1–12)
30-day readmission*	1	0	1

Participants could experience more than one complication; therefore, Clavien–Dindo grade counts represent recorded complication events rather than individual participants

*Unrelated to standard care, e.g. trial of void

## Discussion

The main findings from this pilot randomised trial were that exercise-based prehabilitation intervention was well attended and adhered to by a small group of patients, leading up to breast, prostate, or colorectal cancer surgery. Although there was a higher than expected adverse event frequency, all were low-grade, and no serious adverse events were observed. Given the small achieved sample and exploratory nature of these analyses, the trial was not able to determine whether the intervention influenced cardiorespiratory fitness, health-related quality of life or fatigue, or whether any observed changes were clinically meaningful.

### Feasibility

Recruitment was challenging, with only a relatively small proportion of the broader surgical population identified as potentially eligible and approached for formal screening (47/312), which reflects the well-recognised challenges of recruiting surgical oncology populations into prehabilitation trials [41] and the additional disruption to research activity during the COVID-19 pandemic. Travel burden, time availability, and short timeframes between study enrolment and surgery were the most common reasons eligible patients declined participation, with many required to travel long distances to attend assessment visits at the centralised testing site. Although centre-based delivery enabled direct supervision and individualisation in this pilot study, the travel burden reported by patients suggests that future programmes, irrespective of location, should investigate whether remote or hybrid supervision can improve accessibility while maintaining appropriate exercise monitoring and intervention fidelity [42, 43]. These barriers are likely to be particularly relevant for patients residing in regional or rural areas, where travel may involve additional financial costs, accommodation requirements, time away from work, and reliance on carers or family support. Short pre-operative timeframes have also been identified by clinicians as a key barrier to prehabilitation implementation within routine cancer care [18]. Among patients approached, approximately half consented to participate, indicating that the centre-based programme was acceptable to some patients but had limited reach within the broader surgical population at our site. Individually supervised centre-based delivery also requires patient travel and dedicated staff and facilities, which may limit scalability. Remote or hybrid delivery may reduce participant burden and broaden access in regional settings, although resource use and cost-effectiveness were not evaluated in this pilot study [18].

The median pre-operative intervention window of 18.5 days and the median of five exercise sessions completed in the intervention group reflect the constrained timeframe typical of surgical oncology pathways and are comparable to other exercise-based prehabilitation trials [44–46]. Importantly, this frequency closely aligned with the intended prescription of one session every 2–3 days, indicating that participants were generally able to engage with the intervention as planned despite the limited pre-operative window. Despite the limited intervention period, attendance, adherence and completeness were 91.3%, 82.6%, and 85.7%, respectively, indicating strong engagement among participants. However, the modest number of supervised sessions, limited opportunity for exercise progression, baseline differences in habitual physical activity, and heterogeneity across cancer types may all have influenced the exploratory clinical outcomes [46].

### Safety

We observed a higher frequency of adverse events than expected during the intervention period, corresponding to approximately one event per 12.6 h of supervised training. Differences in adverse event definitions, surveillance intensity, and reporting denominators across studies preclude meaningful direct comparison of event frequencies. Importantly, no serious adverse events occurred, which is consistent with observations from supervised exercise interventions in surgical oncology settings. Contemporary evidence indicates that the harms profile of exercise interventions in adult cancer populations remains uncertain, largely reflecting methodological limitations, inconsistent adverse event reporting, and imprecision of available data [47]. The prescribed exercise intensity did not exceed vigorous levels, consistent with typical safety-oriented prehabilitation prescriptions and partly explaining the absence of serious adverse events. While this conservative intensity approach prioritises tolerability, improving baseline fitness and risk mitigation within a surgical population, higher-intensity strategies such as high-intensity interval training have been proposed as time-efficient alternatives, with multiple reviews demonstrating improvements in selected physiological and functional outcomes alongside reduced time burden [48]. Collectively, these findings align with established exercise oncology guidance indicating that the benefits of exercise generally outweigh the manageable risk of minor adverse events when intensity is appropriately controlled and monitoring is in place [49].

### Exploratory clinical outcomes

The achieved sample was substantially smaller than planned, and the study was not adequately powered to evaluate intervention effectiveness. The exploratory outcome estimates were imprecise and should not be interpreted as demonstrating either benefit or absence of benefit. Previous studies have shown prehabilitation to preserve cardiorespiratory fitness during the pre-operative period compared to usual care [13, 46, 50]. These findings are largely derived from studies involving mostly homogeneous cancer populations, multimodal interventions (e.g. exercise combined with nutritional support), combined exercise modalities (e.g. aerobic and resistance training), and/or the use of estimated rather than directly measured peak V̇O_2_ [4, 14, 51–54]*.* Furthermore, cardiorespiratory fitness-related outcomes were frequently secondary or exploratory and, therefore, often underpowered to detect physiological changes.

The short pre-operative intervention window provided limited opportunity to progress exercise intensity. Participants commenced training at 40% HRR, and some completed relatively few sessions before progression occurred. Given these constraints, future studies could evaluate exercise strategies that provide a greater physiological stimulus within short pre-operative timeframes. High-intensity interval training may also provide a time-efficient alternative and has demonstrated feasibility and improvements in selected physiological and functional outcomes in oncology and prehabilitation settings [45, 48, 55, 56]. However, much of this evidence derives from interventions longer than the pre-operative window available in the present study, and its applicability to very short surgical pathways remains uncertain [45, 55, 56]. To our knowledge, limited studies have examined changes in objectively measured cardiorespiratory fitness across the peri-operative period [41, 57]. Although changes in some peak exercise markers were observed at 30 days after surgery (i.e. peak V̇O_2_ and peak workload), the small sample and imprecise estimates preclude firm conclusions regarding intervention effects or the magnitude of post-operative deconditioning.

Baseline moderate-to-vigorous physical activity was notably higher in the usual care group than in the intervention group. Although baseline cardiorespiratory fitness was similar between groups, this imbalance, along with other differences in baseline characteristics including body mass and the proportion of patients who received neoadjuvant chemotherapy, adds further uncertainty to the interpretation of the exploratory between-group findings.

### Health-Related Quality of Life

While exercise interventions are frequently associated with improvements in health-related quality of life, effects are variable, and short-duration pre-operative programmes may not be sufficient to elicit significant changes [58–60]. In the present study, both groups maintained physical well-being, fatigue, and overall health-related quality of life from T1 to T2; however, the small sample precludes firm interpretation of these changes. Longer-duration programmes, particularly those incorporating resistance training or multimodal components, appear more effective in improving health-related quality of life outcomes [58, 61, 62]. Given the small sample, observed changes in individual health-related quality of life domains were imprecise and should not be interpreted as intervention effects. As sessions were delivered individually, regular contact with an exercise professional may have provided reassurance or support independently of the exercise itself. Conversely, the travel, time commitment, and disruption to daily routines associated with centre-based participation may have negatively influenced well-being [18]. These potential influences were not measured and cannot be separated from the effects of exercise in this pilot study. However, interpretation of health-related quality of life findings should consider the suitability of the FACT-G instrument within a pre-treatment surgical context, as it was originally developed and validated in patients undergoing or having completed cancer treatment [33, 63]. Qualitative research suggests that patients preparing for surgery often prioritise constructs such as preparedness, sense of control, and active agency in recovery, which may not be fully captured by standard health-related quality of life questionnaires [64, 65].

### Limitations

This trial has several limitations. Recruitment and attendance were substantially affected by the COVID-19 pandemic, with the prolonged interruption and associated research restrictions preventing achievement of the original recruitment target of 50 participants. Consequently, the study was underpowered for effectiveness analyses, and the imprecise exploratory clinical outcome estimates cannot support firm conclusions regarding intervention benefit or absence of benefit. The modest intervention exposure, comprising a median of five supervised aerobic sessions within a median pre-operative window of 18.5 days, also provided limited opportunity for exercise progression or physiological adaptation. In addition, formal a priori feasibility thresholds for determining progression to a definitive effectiveness trial were not specified, limiting objective interpretation of whether the pilot findings supported progression.

The single-centre design limits the applicability of these findings to healthcare services with different patient populations, referral pathways, staffing, and resources. Although the inclusion of individuals with prostate, breast, and colorectal cancers was a pragmatic approach for evaluating feasibility within a regional service, differences in disease pathways, surgical procedures, peri-operative care, and expected post-operative outcomes introduced additional variability and limit interpretation of the pooled exploratory clinical outcomes. General physical activity advice provided through multidisciplinary care may also have influenced habitual physical activity and cardiorespiratory fitness. These factors reinforce the need for adequately powered, tumour-specific trials or stratified designs when evaluating clinical effectiveness.

## Conclusion

Among the small number of participants who commenced supervised aerobic prehabilitation, attendance and adherence were favourable, and no serious adverse events were observed. However, the recruitment target was not achieved, and the single-centre, heterogeneous sample limits interpretation of these feasibility and safety observations. As a pilot study with exploratory clinical outcomes, the trial was not adequately powered to determine intervention effects on clinical effectiveness or post-operative outcomes. Future studies should consider broader referral pathways, flexible delivery approaches, and adequately powered tumour-specific or stratified designs. Further research should also examine exercise intensity and progression strategies that can provide an adequate physiological stimulus within short pre-operative timeframes.

## Supplementary Information

Below is the link to the electronic supplementary material.

ESM 1 (DOCX 20.1 KB)

ESM 2 (DOCX 37.8 KB)

## Data Availability

No datasets were generated or analysed during the current study.

## Declarations

### Registration

This randomised controlled trial was prospectively registered on the 14/02/2019 in the Australian New Zealand Clinical Trials Registry (ANZCTR) (ACTRN12619000214134).
