# Supplementary material for: Feasibility and safety of an exercise-based prehabilitation intervention before cancer surgery: a pilot randomised controlled trial
Source: Support Care Cancer. 2026 Oct 21;34(11):1132. doi: 10.1007/s00520-026-11356-9 (PMC13687205; doi:10.1007/s00520-026-11356-9)
Supplement: Supplementary file 1 [file 520_2026_11356_MOESM1_ESM.docx]

**Supplementary Material 1**: Standardised effect sizes (Hedges’ g) with 95% confidence intervals for exploratory CPET-derived outcomes

| Variable | *UCARE (mean ± SD)* | | | *PREHAB (mean ± SD)* | | | Effects (P) | | | Hedges’ g (95% CI) Between Groups | | |
| --- | --- | --- | --- | --- | --- | --- | --- | --- | --- | --- | --- | --- |
|  | T1 (*n*= 8) | T2 (*n*= 8) | T3 (*n*= 7) | T1 (*n*= 11) | T2 (*n*= 11) | T3 (*n*= 6) | Group | Time | Group × Time | T1 | T2 | T3 |
| Time to VT (s) | 773 ± 215 | 833 ± 250 | 697 ± 114 | 741 ± 201 | 759 ± 115 | 593 ± 81 | .18 | .07 | .85 | -0.15 (−1.06 to 0.77) | -0.38 (−1.30 to 0.54) | -0.96 (−2.11 to 0.19) |
| Absolute VT (L/min) | 1.2 ± 0.5 | 1.2 ± 0.5 | 1.1 ± 0.3 | 1.1 ± 0.4 | 1.1 ± 0.3 | 0.8 ± 0.2 | .16 | .22 | .90 | -0.34 (−1.25 to 0.58) | -0.23 (−1.14 to 0.68) | -0.77 (−1.90 to 0.36) |
| Relative VT (mL.kg^-1^.min^-1^) | 12.8 ± 4.5 | 12.7 ± 4.0 | 11.4 ± 2.6 | 12.8 ± 4.2 | 13.4 ± 4.0 | 10.4 ± 3.3 | .94 | .29 | .85 | 0.01 (−0.90 to 0.92) | 0.15 (−0.76 to 1.06) | -0.31 (−1.41 to 0.79) |
| Time to Peak (s) | 996 ± 181 | 1075 ± 249 | 984 ± 143 | 965 ± 149 | 1038 ± 98 | 792 ± 163 | .07 | **.03** | .34 | -0.19 (−1.10 to 0.73) | -0.20 (−1.11 to 0.71) | -1.17 (−2.35 to 0.01) |
| Absolute Peak V̇O_2_ (L/min) | 1.7 ± 0.5 | 1.7 ± 0.5 | 1.5 ± 0.5 | 1.6 ± 0.4 | 1.6 ± 0.3 | 1.1 ± 0.4 | .15 | **.04** | .58 | -0.24 (−1.15 to 0.68) | -0.14 (−1.05 to 0.77) | -0.83 (−1.97 to 0.30) |
| Relative Peak V̇O_2_ (mL.kg^-1^.min^-1^) | 18.0 ± 5.1 | 17.6 ± 4.2 | 15.6 ± 2.6 | 19.1 ± 5.0 | 19.3 ± 5.0 | 14.0 ± 5.2 | .76 | .06 | .59 | 0.22 (−0.70 to 1.13) | 0.35 (−0.56 to 1.27) | -0.38 (−1.48 to 0.72) |
| Peak RER | 1.09 ± 0.13 | 1.13 ± 0.12 | 1.17 ± 0.6 | 1.13 ± 0.8 | 1.16 ± 0.12 | 1.11 ± 0.11 | .87 | .50 | .44 | 0.38 (−0.54 to 1.30) | 0.21 (−0.70 to 1.13) | -0.58 (−1.69 to 0.54) |
| VE/VCO_2_ Slope | 29.2 ± 3.7 | 28.6 ± 3.4 | 29.9 ± 2.8 | 32.3 ± 3.6 | 32.2 ± 4.8 | 33.6 ± 3.5 | **.01** | .61 | .96 | 0.79 (−0.16 to 1.73) | 0.81 (−0.14 to 1.76) | 1.10 (−0.07 to 2.27) |
| Peak Workload (W) | 113.8 ± 31.1 | 116.3 ± 30.7 | 100 ± 23.1 | 104.5 ± 22.5 | 117.3 ± 22.4 | 75 ± 24.3 | .14 | **.01** | .38 | -0.33 (−1.25 to 0.58) | 0.04 (−0.87 to 0.95) | -0.98 (−2.14 to 0.17) |
| Peak RPP | 26062 ± 3963 | 25563 ± 3497 | 25557 ± 2938 | 25224 ± 5169 | 23152 ± 4734 | 25149 ± 8169 | .41 | .70 | .83 | -0.17 (−1.08 to 0.74) | -0.54 (−1.47 to 0.39) | -0.06 (−1.15 to 1.03) |

Abbreviations: VT, Ventilatory Threshold; CPET, Cardiopulmonary Exercise Test; V̇O_2_, Maximal Oxygen Consumption; VCO_2_, Carbon Dioxide Output; W, watts; RER, Respiratory Exchange Ratio; RPP, Rate Pressure Product,
